# Context matters: measuring implementation climate among individuals and groups

**DOI:** 10.1186/1748-5908-9-46

**Published:** 2014-04-17

**Authors:** Sara R Jacobs, Bryan J Weiner, Alicia C Bunger

**Affiliations:** 1 Department of Health Policy and Management, Gillings School of Global Public Health, 1101 McGavran-Greenberg Hall, CB #7411, University of North Carolina at Chapel Hill, Chapel Hill, NC 27599-7411, USA; 2 Cecil G. Sheps Center for Health Services Research, 725 Martin Luther King Jr. Blvd., CB #7590, University of North Carolina at Chapel Hill, Chapel Hill, NC 27599-7411, USA; 3 College of Social Work, The Ohio State University, 325W Stillman Hall, 1947 College Road, Columbus, OH 43210, USA

**Keywords:** Implementation climate, Organizational context, Measurement of global constructs, Measurement of group level phenomenon

## Abstract

**Background:**

It has been noted that implementation climate is positively associated with implementation effectiveness. However, issues surrounding the measurement of implementation climate, or the extent to which organizational members perceive that innovation use is expected, supported and rewarded by their organization remain. Specifically, it is unclear whether implementation climate can be measured as a global construct, whether individual or group-referenced items should be used, and whether implementation climate can be assessed at the group or organizational level.

**Methods:**

This research includes two cross-sectional studies with data collected via surveys at the individual level. The first study assessed the implementation climate perceptions of physicians participating in the National Cancer Institute’s (NCI) Community Clinical Oncology Program (CCOP), and the second study assessed the perceptions of children’s behavioral health clinicians implementing a treatment innovation. To address if implementation climate is a global construct, we used confirmatory factor analysis. To address how implementation climate should be measured and at what level, we followed a five-step framework outlined by van Mierlo and colleagues. This framework includes exploratory factor analysis and correlations to assess differences between individual and group-referenced items and intraclass correlations, interrater agreements, and exploratory factor analysis to determine if implementation climate can be assessed at the organizational level.

**Results:**

The confirmatory factor analysis demonstrated that implementation climate is a global construct consisting of items related to expectations, support and rewards. There are mixed results, however, as to whether implementation climate should be measured using individual or group-referenced items. In our first study, where physicians were geographically dispersed and practice independently, there were no differences based on the type of items used, and implementation climate was an individual level construct. However, in the second study, in which clinicians practice in a central location and interact more frequently, group-referenced items may be appropriate. In addition, implementation climate could be considered an organizational level construct.

**Conclusions:**

The results are context-specific. Researchers should carefully consider the study setting when measuring implementation climate. In addition, more opportunities are needed to validate this measure and understand how well it predicts and explains implementation effectiveness.

## Background

Implementation climate has emerged as an important construct in implementation science. It first appeared in 1996 in Klein and Sorra’s theory of innovation implementation, where the authors defined it as organizational members’ ‘shared summary perceptions of the extent to which their use of a specific innovation is rewarded, supported, and expected within their organization’ [1]. According to the theory, implementation climate, along with fit between the innovation and the organizational members’ values, are key predictors regarding the consistency and quality of organizational members’ use of a specific innovation [1]. Sustained and systematic use of an innovation can determine the ultimate benefits an organization receives by implementing a given innovation (*e.g*., increased profitability, employee morale, productivity) [1]. Implementation climate has subsequently been discussed in a landmark systematic review of the diffusion of innovations in health services research [2] and has also been incorporated into the Consolidated Framework for Implementation Research [3]. Although empirical research has lagged behind theoretical discussion, the construct has been considered in several studies of implementation in health and human service agencies, schools, and manufacturing organizations [4-11]. Evidence from this mix of qualitative and quantitative studies finds that implementation climate is linked to consistent, high-quality innovation use, as Klein and Sorra predicted. In a recent commentary, we noted that the construct has the potential to bring theoretical and empirical coherence to a growing body of research on organizational barriers and facilitators of effective implementation [12].

Although implementation climate has garnered theoretical attention and empirical support, no standard approach for measuring the construct exists. In prior work, we identified three measurement issues that hinder efforts to establish construct validity, compare results across studies, and accumulate scientific knowledge [12]. First, can implementation climate be measured as a global construct? Klein and Sorra postulate that implementation climate reflects organizational members’ gestalt perceptions of the expectations, support and rewards for innovation use [1]. Although some studies have used factor analysis to determine whether the three dimensions of implementation climate together form a global construct [4,6], these studies have used very specific items related to information systems implementation (*e.g*., ‘help desk’ availability) that have questionable relevance for implementation research in health and human services. Other studies have examined implementation climate dimensions separately [5] rather than as a global construct or have measured implementation climate with items that do not reflect the three dimensions [13].

Second, should implementation climate be measured with individually referenced items or group-referenced items? Climate researchers disagree about whether climate constructs are better measured as the aggregation of individuals’ perceptions of their own experience (*e.g*., ‘I am expected to use the innovation’) or individuals’ perceptions of collective experience (*e.g*., ‘We are expected to use the innovation’) [12]. Some scholars contend that individually referenced items encourage respondents to look within and ignore collective experience [4,5] while others argue that respondents are more accurate judges of their own experience than the experience of the group [12,14]. To date, studies of implementation climate have used group-referenced items [4-6,13]; however, studies involving other climate constructs in implementation research — such as Glisson’s Organizational and Social Context — have used individually referenced items [15-19]. Some evidence suggests that individually referenced items and group-referenced items measure distinct constructs [20,21]; however, it is unknown whether this distinction makes a difference in the measurement of implementation climate.

Lastly, can implementation climate be reliably and validly measured as an organization-level construct? Klein and Sorra regard implementation climate as a ‘shared team property’, meaning that organizational members share sufficiently similar perceptions of implementation climate and that those perceptions can be characterized as a whole [1]. Their theory is pitched at an organizational level, whereby organizational members’ shared perceptions of the expectations, support and rewards for innovation use determines the overall, pooled or aggregate consistency and quality of innovation use. However, prior studies offer limited evidence that implementation climate can be reliably and validly measured as an organization-level construct using aggregated data collected from individuals. Some studies have not aggregated implementation climate perceptions to the organizational level [5,6] and, in some cases, group-referenced items were used to explain individual-level behavior [5,6]. Since implementation climate is conceived as an organization-level construct, it is important to verify that sufficient within-group agreement exists to justify aggregating individuals’ climate perceptions to the organizational level of analysis.

Therefore, the goal of this study was to examine three aspects regarding the measurement construct of implementation climate. Given the previous work on implementation climate, we hypothesized that implementation climate is a global construct, composed of questions relating to expectations, support and rewards, which could be assessed at the organizational level [1,4-12]. We also believed using group-referenced items would be more reliable than individually referenced items given the focus on shared perceptions of climate [12]. Although additional research is required, we sought to provide initial evidence regarding three aspects of measuring implementation climate by examining them within two distinct health services settings.

## Methods

We developed a brief instrument consisting of items that were specific enough to capture the three dimensions of implementation climate, yet generic enough to apply broadly to implementation of health and human service innovations. We then fielded the instrument in two different study settings because theory and research suggest that the measurement of implementation climate might be sensitive to features of the organizational and social context of implementation [1,12,20]. These two settings provided a distinct set of organizational and environmental factors to examine implementation climate. Our results indicate that implementation climate can be measured as a global construct; however, the importance of reference-group wording of survey items and the reliability and validity of the implementation climate as an organization-level construct are somewhat dependent on context.

### Measures

Using Klein and Sorra’s [1] original definition of implementation climate, we created an instrument consisting of six items, two items per climate dimension (*i.e*., expected, supported and rewarded). We generated the items by reviewing the literature for existing items, consulting program officials, and drawing on prior studies of CCOP and learning collaboratives for substantive content, and following published guidelines for measuring organizational constructs generally and implementation climate specifically. A survey methodologist provided an expert review of item wording, item ordering, response options, survey length, cognitive burden, social desirability, and survey formatting. Table 1 lists the items used in the two studies. We kept item wording as consistent as possible across the studies, but permitted some tailoring to account for differences in study participants, interventions, and implementation context. In both studies, the items were phrased first as individually referenced items, and then repeated as group-referenced.

**Table 1:** Exploratory factor analysis results

					**Factor loadings**		
**Item**	**Item wording**	**N**	**Mean**	**Standard deviation**	**I**	**II**	**III**
*Study 1 results*							
Q1	I am expected to enroll a certain number of patients in NCI clinical trials.	47	3.37	1.05	-0.04	0.01	-0.01
Q2	I am expected to help the CCOP meet its clinical trial enroll.	47	4.20	1.09	0.02	0.04	0.70
Q3	I get research support to identify potentially eligible patients for NCI clinical trials.	47	3.79	1.25	-0.01	0.93	0.03
Q4	I get research support to enroll patients in NCI clinical trials.	47	4.08	1.17	0.05	0.95	-0.05
Q5	I receive recognition when I enroll patients in NCI clinical trials.	47	3.17	1.31	0.98	0.02	-0.01
Q6	I receive appreciation when I enroll patients in NCI clinical trials.	47	3.27	1.28	0.70	0.04	-0.01
Q7	Physicians are expected to enroll a certain number of patients in NCI clinical trials.	47	3.46	1.35	0.04	-0.01	0.02
Q8	Physicians are expected to help the CCOP meet its clinical trial enroll.	47	4.18	0.98	0.03	-0.03	0.70
Q9	Physicians get support to identify potentially eligible patients for NCI clinical trials.	47	3.81	1.08	0.04	0.77	-0.03
Q10	Physicians get support to enroll patients in NCI clinical trials.	47	3.96	1.08	0.05	0.80	0.05
Q11	Physicians receive recognition when I enroll patients in NCI clinical trials.	47	3.27	1.23	0.96	0.03	0.02
Q12	Physicians receive appreciation when I enroll patients in NCI clinical trials.	47	3.37	1.19	0.69	0.06	0.01
*Study 2 results*							
Q1	I am expected to use TF-CBT with a certain number of clients.	26	3.74	0.83	0.07	-0.05	0.28
Q2	I am expected to help my agency meet its goals for implementing TF-CBT.	26	4.32	0.63	-0.01	0.03	0.88
Q3	I get the support I need to identify potentially eligible clients for TF-CBT.	26	4.20	0.89	0.92	0.09	0.14
Q4	I get the support I need to use TF-CBT with my clients.	26	4.14	0.87	0.89	-0.02	-0.12
Q5	I receive recognition when I use TF-CBT with my clients.	26	3.38	0.73	0.20	0.42	0.08
Q6	I receive appreciation when I use TF-CBT with my clients.	26	3.19	0.81	0.14	0.79	-0.07
Q7	Clinicians are expected to use TF-CBT with a certain number of clients.	26	3.68	0.89	-0.07	0.15	0.40
Q8	Clinicians are expected to help our agency meet its goals for implementing TF-CBT.	26	4.15	0.55	0.11	0.01	0.82
Q9	Clinicians get the support they need to identify potentially eligible clients for TF-CBT.	26	4.06	0.87	0.38	-0.07	0.23
Q10	Clinicians get the support they need to use TF-CBT with eligible clients.	26	3.92	1.07	0.05	0.12	-0.13
Q11	Clinicians receive recognition for using TF-CBT with eligible clients.	26	3.39	0.81	0.03	0.76	0.06
Q12	Clinicians receive appreciation for using TF-CBT with eligible clients.	26	3.25	0.92	-0.20	1.01	0.05

### Study setting and sample

Study 1. The National Cancer Institute’s (NCI) Community Clinical Oncology Program (CCOP) is a provider-based research network that conducts clinical trials in community-based practice settings and translates research results into clinical practice. The NCI’s CCOP network is a three-way partnership involving the NCI’s Division of Cancer Prevention (NCI/DCP), selected cancer centers and clinical cooperative groups (CCOP Research Bases), and community-based network hospitals and physician practices (CCOP Organizations) [22-26]. NCI/DCP provides overall direction and funding for community hospitals and physician practices to participants in clinical trials in cancer treatment, prevention and control; CCOP Research Bases design the clinical trials and analyze the results; and CCOP Organizations enroll patients, collect data, and disseminate study findings [22]. As of April 2013, there are 47 CCOP Organizations across 28 states, the District of Columbia, and Puerto Rico. The CCOP network includes over 450 hospitals and more than 2,700 physicians. On average, CCOP organizations have 10 participating community hospitals and physician practices, and 48 participating oncologists, surgeons, and other physicians.

We obtained data on CCOP physicians’ perceptions of implementation climate for conducting clinical trials in community practice settings (an innovation for community-based physicians) through a survey administered in the fall of 2011. The sampling frame included all CCOP-affiliated physicians eligible to enroll patients in clinical trials. Responses were collected between October 2011 and January 2012. One week after sending potential respondents a postcard announcing the survey and highlighting its importance to NCI, physicians were sent a cover letter explaining the goals of the survey, the survey itself, a self-addressed and stamped return envelope, and a $50 Visa gift card as an incentive to complete the survey. Physicians were also able to complete the survey online via a unique access code provided in the mailings. A thank you or reminder postcard was then sent the following week. Approximately three weeks after the first mailing, non-respondents received a second copy of the survey, cover letter, and return envelope. Lastly, we contacted CCOP PIs and CCOP Administrators to email the non-responding physicians affiliated with their CCOP requesting them to complete the survey. This study was approved by the Institutional Review Board at the University of North Carolina at Chapel Hill.

Study 2. In 2011, youth-serving agencies in a medium-sized Midwestern city were invited to participate in an initiative led by the county government to implement trauma-focused cognitive behavioral therapy (TF-CBT). TF-CBT is a widely disseminated manualized treatment with strong evidence supporting its effectiveness for reducing post-traumatic stress disorder symptoms among children and youth [27-29]. The invited agencies sent implementation teams (3 to 10 employees) to a year-long TF-CBT Learning Collaborative, an adaptation of the Institute for Healthcare Improvement’s Breakthrough Series Collaborative [30,31]. To accommodate agency interest, four Learning Collaboratives were conducted.

The Learning Collaboratives brought participants together for three face-to-face learning sessions. We obtained data on implementation team members’ perceptions of implementation climate through a paper-based survey administered in person during the third and final learning session of each of the four Collaboratives. At the beginning of the third learning session, a member of the research team gave an overview of the study, obtained consent, and distributed the survey to the team members present, and subsequently collected completed surveys. Responses were collected from January through September 2012. This study was approved by the Institutional Review Boards at the Ohio State University and the University of North Carolina at Chapel Hill.

### Data analysis

**Figure 1:**
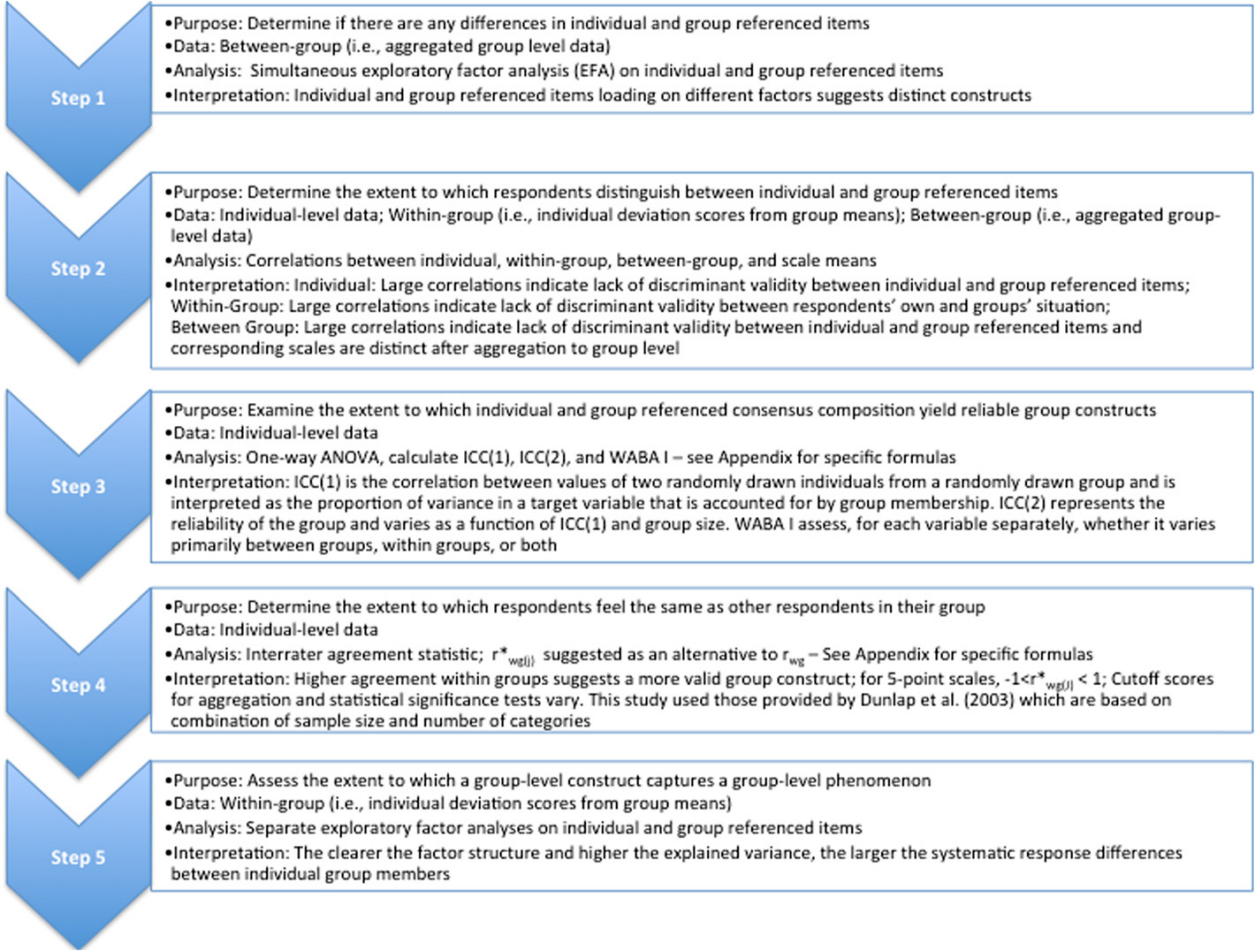
Five-step process to determine group-level construct from individual data.

To determine whether implementation climate can be measured as a global construct, we used confirmatory factor analysis (CFA) to ascertain whether implementation climate is comprised of three factors (expected, supported and rewarded). To assess whether implementation climate should be measured by individually referenced items or group-referenced items and as an organization-level construct, we employed van Mierlo and colleagues’ five-step procedure for composing group-level constructs from individual-level survey data [32] (Figure 1). Analysis details are discussed in detail within the results section. All analyses were conducted in Stata 12 [33].

## Results

### Response rates and participant characteristics

Study 1. The survey was sent to a stratified random sample of 817 physicians. On average, 17 physicians were surveyed per CCOP, and 10 physicians responded per CCOP organization. We obtained a total response rate of 63% (N = 485). No significant differences were observed between survey respondents, non-respondents, and CCOP physicians (N = 2,725) in physician age, practice type (*e.g*., group practice), training location, medical specialty, or gender. A total of 74% were male; 26% were female; 75% were Caucasian, non-Hispanic; 15% were Asian; and the remaining 10% were either African-American, Native Hawaiian/Pacific Islander, or reported multiple races.

Study 2. Of the 155 team members from 26 behavioral health agencies that participated in the final learning session, 137 responded (88% response rate). Most respondents were direct service clinicians (63%), followed by supervisors (21%) and senior leaders (14%). Nearly all (94%) held a master’s degree or higher in social work (51%), counseling (15%), psychology (12%), or other helping profession. Respondents had extensive experience serving families and children, with 64% reporting five years’ or more experience. However, many participants were new to their agencies; 42% were employed at their agency for a year or less at the time they began participating in the Collaborative.

### Implementation climate as a global construct

**Figure 2:**
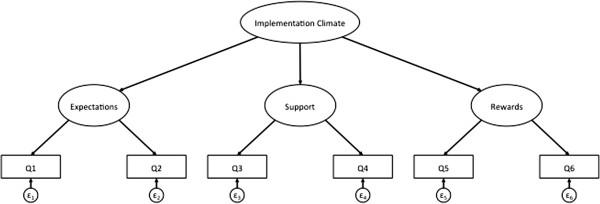
Example of second order CFA model for individually referenced items.

**Figure 3:**
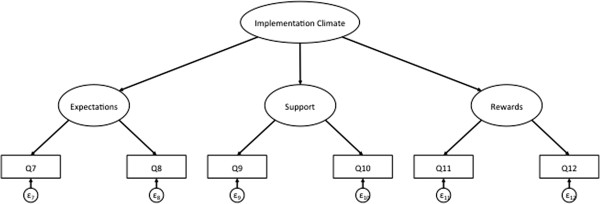
Example of second order CFA model for group-referenced items.

To determine if implementation climate can be measured as global construct consisting of expectations, rewards and support, we ran a series of CFAs to fit a second-order factor structure to the data in each study, with separate analyses for the individually and group-referenced items (Figures 2 and 3). In both studies, the second-order CFA model for the individually referenced items converged and demonstrated strong fit (Table 2). Based on model fit and the resulting modifications indices (*i.e*., the minimum that the chi-square statistic is expected to decrease if the corresponding parameter is no longer assumed to be fixed at zero), we did not need to make any post-hoc modifications to the model in either study [34-36].

**Table 2:** Confirmatory factor analysis results

	**Study 1: CFA standardized factor loading**	**Study 2: CFA standardized factor loading**
Individual referenced items: observed variables		
Q1	0.563 (0.061)	0.821 (0.081)
Q2	0.948 (0.087)	0.597 (0.078)
Q3	0.894 (0.029)	0.851 (0.040)
Q4	0.853 (0.029)	0.953 (0.036)
Q5	0.9050 (.029)	0.932 (0.031)
Q6	0.862 (0.029)	0.895 (0.033)
Individual referenced items: latent variables		
Expectations	0.457 (0.067)	0.717 (0.089)
Support	0.743 (0.067)	0.757 (0.071)
Rewards	0.695 (0.063)	0.859 (0.068)
Group referenced items: observed variables		
Q7	0.597 (0.060)	0.860 (0.052)
Q8	0.854 (0.075)	0.941 (0.052)
Q9	0.845 (0.028)	0.808 (0.059)
Q10	0.900 (0.028)	0.943 (0.061)
Q11	0.922 (0.025)	0.916 (0.035)
Q12	0.850 (0.026)	0.946 (0.034)
Group referenced items: latent variables		
Expectations	0.458 (0.063)	0.637 (0.097)
Support	0.664 (0.063)	0.688 (0.103)
Rewards	0.836 (0.071)	0.736 (0.098)

Standard Error in parenthesis.

Study 1 Individual Referenced: *X*2 = 0.2391 CFI = 0.998; TLI = 0.996; SRMR = 0.015; RMSEA = 0.027.

Study 1 Group Referenced: *X*2 = 0.1146 CFI = 0.997; TLI = 0.990; SRMR = 0.013 RMSEA = 0.040.

Study 2 Individual Referenced: *X*2 = 0.6650 CFI = 1.00; TLI = 1.01; SRMR = 0.012; RMSEA = 0.00.

Study 2 Group Referenced: *X*2 = 0.629 CFI = 0.996; TLI = 0.983; SRMR = 0.013; RMSEA = 0.065.

For the group-referenced items in both studies, the model fit and modifications indices indicated that we needed to make post-hoc modifications. CFA is an iterative process in which model fit is improved by using theory and modifications indices either to add additional pathways between variables or to allow items to co-vary [34-36]. For example, items included may share common variation that is not explained by any of the proposed relationships in the model. Therefore, post-hoc modifications were only added if they could be theoretically justified and improved model fit.

In Study 1, we allowed the error terms of the following group-referenced items to co-vary higher than with other variables: ‘Physicians are expected to enroll a certain number of patients in NCI-sponsored clinical trials’, and ‘Physicians get the research support they need to enroll patients in NCI-sponsored clinical trials’. We hypothesized that these items might co-vary because CCOPs with formal expectations for minimum enrollment are likely to provide more support to enroll patients. In addition, larger, more mature CCOPs may be more likely to institute expectations and have more resources to offer more support for enrollment. For Study 2, we allowed the error terms of the following group-referenced items to co-vary higher than with other variables: ‘Clinicians are expected to use TF-CBT with a certain number of clients,’ to co-vary with the error terms for the items, ‘Clinicians are expected to help our agency meet its goal for implementing TF-CBT,’ and ‘Clinicians get support they need to use TF-CBT with eligible clients.’ We hypothesized that these items might co-vary because agency goals for implementation were closely tied to individual clinicians’ ability to use TF-CBT with at least five clients in order to be eligible for inclusion on a local roster of trained TF-CBT clinicians.

In both studies, CFA results indicate that implementation climate can be measured as a global construct using either individually referenced items or group-referenced items. We generated global implementation climate scales using the standardized factor loading from the second-order CFA models. For example, in constructing each scale, we weighted each item based on its standardized factor loading before taking the average across all items. These global scales are used in Steps 2 to 4 in the analysis below.

### Measurement of implementation climate

#### *Step 1. Similarity of constructs through factor analysis between groups*

The goal of Steps 1 and 2 was to determine whether individual and group referenced items measure the same construct. The extent to which the individually referenced and group-referenced items yield distinct group-level constructs was examined with exploratory factor analysis (EFA) of group-level data (47 CCOPs in Study 1; 26 teams in Study 2). It is important to conduct the factor analysis on the group level data because a structure in which the individually and group-referenced items systematically load on different components indicates that they represent distinct group constructs [32]. In Study 1, group-level EFA yielded three factors explaining 94% of the variance. Individually referenced items that load in a block on a different factor from group-referenced items indicate that the items measure different constructs. However, following oblique rotation, the individually referenced and group-referenced items loaded in pairs on the same factor (Table 1). For example, the two individually referenced items measuring the ‘rewarded’ dimension of implementation climate loaded highly on the first factor, as did the two group-referenced items measuring the same dimension. Only one individually referenced item measuring the ‘expected’ dimension of implementation climate and its corresponding group-referenced item loaded highly on the third factor. The other individually referenced item measuring the ‘expected’ dimension and its corresponding group-referenced item did not load highly on any of the first three factors in the EFA solution. The factor loadings for the pair of ‘expected’ items may have diverged because most CCOPs did not set minimum enrollment requirements for individual physicians. Overall, the group-level EFA results in Study 1 do not indicate that individually referenced and group-referenced items are measuring different constructs.

In Study 2, group-level EFA also yielded three factors explaining 89% of the variance (Table 1). Individually referenced and group-referenced items did not load in a block on different factors. Instead, items exhibited a mixed pattern of factor loadings even after oblique rotation. For example, both individually referenced items measuring the ‘supported’ dimension of implementation climate loaded highly onto the first factor, yet neither of the corresponding group-referenced items loaded onto any factor. Like Study 1, Study 2 results do not indicate that individually referenced and group-referenced items are measuring different constructs.

#### *Step 2. Similarity of constructs through correlations*

We further explored differences between individually referenced and group-referenced items with correlations, where small correlation coefficients indicate that individually referenced and group-referenced items measure distinct constructs. Vice versa, larger correlations indicate that individual and group-referenced items are measuring the same construct. The correlations between individually referenced and group-referenced items among individuals indicate the extent to which individuals differentiate their own situation from that of the other individuals within their CCOP or team. Correlations at the individual-level can be distorted, however, because they do not account for the clustering in the data structure. The correlations between individually referenced and group-referenced items within groups (*i.e*., individual deviations from group means) correct for clustering and indicate the extent to which individuals distinguish their individual perception of their own situation from that of their group situation as a whole, rather than other physicians or clinicians within the group. Lastly, the correlation of individually referenced and group-referenced items between groups indicates the extent to which these items are distinct after aggregating the individual responses to the group level.

For Study 1, the correlations for each item pair and for the scale means at the individual, within-group, and between-group levels are moderately high, indicating a relatively high degree of shared variance between the individually referenced and group-referenced measures (Table 3). Focusing on the scale means, the percentage of shared variance between individually referenced and group-referenced measures of implementation climate was 69% at the individual level (r = 0.82, p <0.01), 67% at the within-group level (r = 0.81, p <0.01), and 79% at the between-group level (r = 0.90, p <0.01). These results corroborate the results of Step 1: individually referenced and group-referenced items do not appear to be measuring different constructs for physicians in the CCOP.

**Table 3:** Correlations between items and scales within and between groups

**Items**	**Study 1: Individual**	**Study 2: Individual**	**Study 1: Within-group**	**Study 2: Withing-group**	**Study 1: Between-group**	**Study 2: Between-group**
Q1 x Q7	0.78	0.53	0.73	0.43	0.92	0.68
Q2 x Q8	0.76	0.71	0.75	0.65	0.86	0.84
Q3 x Q9	0.75	0.59	0.74	0.31	0.80	0.83
Q4 x Q10	0.75	0.55	0.74	0.40	0.84	0.64
Q5 x Q11	0.81	0.78	0.79	0.76	0.92	0.74
Q6 x Q12	0.78	0.76	0.78	0.72	0.78	0.82
Scale average	0.83	0.66	0.82	0.51	0.89	0.83
Percentage of shared variance	69%	44%	67%	26%	79%	69%

Study 1: *N*_*individual*_ and *N*_*within*_ = 470; *N*_*between*_ = 47.

Study 2: *N*_*individual*_ and *N*_*within*_ = 135; *N*_*between*_ = 26.

All correlations significant at p < .01.

For Study 2, the correlations for each pair and the scale means are much lower than in Study 1, especially for individual and within-group correlations. Focusing on the scale means, the percentage of shared variance between individually referenced and group-referenced measures of implementation climate was only 44% at the individual level (r = 0.66, p <0.01), 26% at the within-group level (r = 0.51, p <0.01), and 69% at the between-group level (r = 0.83, p <0.01). In contrast to Study 1, Study 2 results suggest individually referenced items and group-referenced items may measure different constructs.

### Implementation climate at the organizational level

#### *Step 3. Construct validity through variance within and between groups analysis*

The goal of the remaining three steps was to assess whether implementation climate can be aggregated to the organizational level to measure implementation climate of the entire organization. Ideally, a reliable organization-level measure should differentiate between organizations. The extent to which individually referenced and group-referenced items produce reliable organization-level constructs was examined by computing two intraclass correlation coefficients — ICC(1) and ICC(2) — from a one-way random-effects analysis of variance. The higher ICC(1) and ICC(2), the greater the extent to which climate perceptions are shared by organizational members and the more reliable the organization-level construct.

In Study 1, the ICCs for the group-referenced implementation climate scale are slightly larger than those for the individually referenced scale: 0.08 versus 0.07 for ICC(1), and 0.48 versus 0.44 for ICC(2) (Table 4). Although there is some clustering in the data, it seems that much of the variance in implementation climate perceptions resides at the individual level. The absolute values for the ICC(2) are also modest in size and fall below the commonly applied 0.80 cutoff [37].

**Table 4:** ICC(1), ICC(2), and WABA I results

	**Intraclass correlations**				**WABA I**		
	**ICC(1)**	**ICC(2)**	**F test**^**a,b**^	**Study 1: ηbetween**	**Study 1: ηwithin**	**E-Test**^**c**^	**1/F Test**^**d**^
Study 1: Individual referenced	0.07	0.44	1.80**	0.40	0.91	0.44	0.55
Study 1: Group referenced	0.08	0.48	2.14**	0.43	0.90	0.48	0.47
Study 2: Individual referenced	0.29	0.66	2.92 **	0.65	0.76	0.86	0.21
Study 2: Group referenced	0.33	0.70	3.37**	0.67	0.74	0.91	0.19

Note: ICC = intraclass correlation coefficient; WABA = within and between analysis **p < .01.

^a^Study 1: df(within) = 423 for individual referenced = 426 for group referenced; df(between) = 46.

^b^Study 2: df(within) = 133 for individual referenced =129 for group referenced; df(between) = 26.

^c^E-test for parts significant at 30°.

^d^A parts condition, in which ηw > ηb requires an inverse F-test (1/F) with df = N – J and J – 1 for the numerator and denominator respectively.

In Study 2, the ICC(1) and ICC(2) values are larger than those in Study 1 (Table 4). Like Study 1, the ICCs for the group-referenced implementation climate scale are slightly larger than those for the individually referenced scale: 0.33 versus 0.29 for ICC(1), and 0.70 versus 0.66 for ICC(2). Although the values for the ICC(2) fall below the 0.80 cutoff, implementation climate is a more reliable organization-level construct among clinicians in children’s behavioral health organizations than for physicians in CCOPs.

#### *Step 4. Construct validity assessment of agreement among group members*

The validity of organization-level constructs was examined by calculating interrater agreement, or the extent to which group members provide identical ratings of implementation climate. Organization-level constructs created from individual-level data have greater validity when group members provide similar ratings in an absolute sense, or at least more similar than random responses [20,38,39]. A common measure of interrater agreement is the r*_wg(J)_ index for multiple items.

In Study 1, the average r*_wg(J)_ values for all 47 CCOPs were 0.74 for the individually referenced implementation climate scale and 0.79 for the group-referenced implementation climate scale (Table 5). A total of 66% of the CCOPs showed a higher r*_wg(J)_ for group-referenced implementation climate scale than for the individually referenced implementation climate scale; however, for most CCOPs, the absolute difference was small, ranging from 0.00 to 0.24 (mean difference = 0.07). Based on Dunlap and colleagues’ [40] suggested significance levels for various combinations of sample size and number of response categories, 89% of CCOPs showed significant r*_wg(J)_ values for individually referenced implementation climate scale, and 94% showed significant r*_wg(J)_ values for the group-referenced implementation climate scale. Study 1 results suggest slightly higher within-group agreement for the group-referenced implementation climate scale compared to the individually referenced implementation climate scale.

**Table 5:** Interrater agreement

	**N**	Individual referenced average r*wg(J)	Percent significant individual referenced r*wg(J)	Group referenced average r*wg(J)	Percent significant group referenced r*wg(J)
Study 1	47	0.74	89%	0.79	94%
Study 2	26	0.73	58%	0.76	62%

In Study 2, the average r*_wg(J)_ values for the 26 implementation teams in the children’s behavioral health agencies were similar to those obtained in Study 1. The average r*_wg(J)_ value was 0.73 for the individually referenced implementation climate scale and 0.76 for the group-referenced implementation climate scale. Unlike Study 1, a slight majority of teams (54%) showed a higher r*_wg(J)_ for the individually referenced implementation climate scale than for the group-referenced implementation climate scale, although the absolute difference was also small (mean difference = 0.16). The teams with the greatest absolute difference tended to have greater r*_wg(J)_ for the group items than the individual items, indicating that members of these teams reported wide variation in their perceptions about their individual experiences, but rated the general experience of clinicians in their agencies similarly. The large discrepancy in r*_wg(J)_ values tended to occur in generalist agencies or those in non-traditional settings. In these settings, it may be more difficult for clinicians to implement TF-CBT. Based on Dunlap and colleagues’ [40] suggested significance levels, only 40% of teams showed significant r*_wg(J)_ values for the individually referenced implementation climate scale, and 60% of teams showed significant r*_wg(J)_ values for the group-referenced implementation climate scale. These percentages are smaller than those obtained in Study 1, perhaps because significance levels vary based on team size. The average team in Study 2 is smaller (mean = 7) than the average CCOP in Study 1. As in Study 1, Study 2 results suggest a slightly higher within-group agreement for the group-referenced implementation climate scale compared to the individually referenced implementation climate scale.

#### *Step 5. Construct validity through factor analysis within groups*

The extent to which organization-level measures capture a group-level phenomenon [41] was examined through a factor analysis of within-group data (*i.e*., individual deviations from the group mean). Although individuals within groups are often more similar to each other than individuals across groups, individuals within groups are not identical to one another, and individually referenced items should still be sensitive to individual differences within a group. A within-group factor analysis should yield a clear one-factor structure if ‘true’ differences in individual perceptions persist after ‘subtracting’ shared perceptions within the group. To the extent that group members make reliable judgments about group-level phenomena (*e.g*., implementation climate of the group), the differences in individual group members’ responses represent measurement error. If that measurement error is non-systematic, a within-group factor analysis of group-referenced items should yield no meaningful structure.

In Study 1, within-group factor analysis yielded a clear one-factor structure for both the individually referenced and group-referenced items (Table 6). These results indicate that within-group component structures for the group-referenced items do not merely represent independent measurement error, as expected. Instead, they reflect systematic individual differences, much like the individually referenced items. In Study 2, however, within-group factor analysis yielded a clear one-factor structure for the individually referenced items, but not for the group-referenced items. Instead, the group-referenced items loaded on a two-factor structure. For Study 2, there may be a meaningful two-factor structure, although what constitutes a ‘meaningful structure’ is unclear in the literature. While van Mierlo and colleagues suggest that a meaningful structure in the group-referenced items may suggest systematic measurement error, we have no reason to believe that our items were assessed with any systematic measurement error. Perhaps the two-factor structure suggests similar variances within the group.

**Table 6:** Factor analysis within groups

	**Study 1: individual referenced**	**Study 2: individual referenced**	**Study 1: group referenced**	**Study 2: group referenced**	
% Variance	80%	91%	82%	66%	29%
Component loadings					
Q1	0.36	0.38			
Q2	0.46	0.25			
Q3	0.71	0.26			
Q4	0.69	0.39			
Q5	0.74	0.80			
Q6	0.72	0.80			
Q7			0.31	0.15	0.84
Q8			0.41	0.20	0.83
Q9			0.69	0.16	0.14
Q10			0.70	0.29	0.00
Q11			0.77	0.86	0.14
Q12			0.75	0.87	0.20

## Discussion

We sought answers to three measurement questions about an important construct in implementation theory and research: can implementation climate be measured as a global construct; should implementation climate be measured with individually referenced or group-referenced items; and can implementation climate be reliably and validly measured at an organizational level? We explored these questions in the context of two studies. The answers to these questions varied somewhat across the two studies, suggesting that, when it comes to the measurement of implementation climate, context matters.

### Measuring implementation climate as a global construct

The results of both studies indicated that implementation climate can be measured as a global construct composed of items reflecting expectations, support and rewards for innovation use. These results are consistent with Klein and Sorra’s [1] conceptualization of implementation climate and consistent with prior research [4,13]. Although no standard instrument exists for measuring implementation climate, the instrument that we developed includes items that reflect all three dimensions of implementation climate and phrases items generally enough to apply in a wide range of contexts. The main difference in factor loadings between the individually referenced items in the two studies is with the items related to expectations. For the CCOP-affiliated physicians, expectations related to helping the CCOP meet its goals were more heavily weighted than the expectations for personal enrollment. This makes sense, as most CCOPs do not institute minimum requirements for patient enrollment; therefore, physicians note the CCOP’s goals as more important in determining an implementation climate. In contrast, the children’s behavioral health clinicians had formal requirements for using TF-CBT and therefore more heavily weighted their individual expectations as compared to expectations for the agency as a whole. This is reflected in the difference in the loadings on the latent variable for expectations as well.

For the CCOP-affiliated physicians, the group-referenced item loadings are similar to the individually referenced items. For the clinicians in Study 2, however, the group-referenced item loadings for expectations are higher than the loadings on the individually referenced items. This is likely because clinicians were better able to assess differences in their own experiences and that of other clinicians. Although they placed less emphasis on helping to meet the agency’s goals when the question was individually referenced, they did believe that clinicians in general are expected to help the agency meet its goals for implementing TF-CBT. This is reflected also in our group-referenced CFA, as clinicians gave more equal weight to expectations aimed at both using TF-CBT with a certain number of clients as well as expected the agency meet its goals, most likely because the agency’s implementation goals are so closely tied to individual clinicians’ ability to using TF-CBT with at least five clients.

Since Klein and Sorra [1] conceived implementation climate as a global construct, a proper test of their theory of implementation effectiveness should occur when a second-order factor structure consisting of expectations, support and rewards fits the data. This condition was met in both of our study settings; however, for studies in which a clear factor structure does not fit, researchers may still advance theory and research. The data might be used to examine whether some climate dimensions explain more variance in implementation effectiveness than others. For example, in some contexts, support for an innovation’s use may be more important than rewards, especially if implementation is mandatory.

### Measuring implementation climate using individually or group-referenced items

The results of the Study 1 suggested that it does not matter whether implementation climate is measured using individually referenced items or group-referenced items. The results of Study 2 suggested that it does matter, at least to some extent. This divergence in study findings is likely due to differences in context.

Group members form perceptions of each other’s experiences through direct observation and verbal communication, both of which are facilitated by social interaction and physical proximity. Physicians participating in CCOPs typically practice in multiple, geographically dispersed locations. On average, CCOPs consist of 10 physician practices and community hospitals. Although physicians practicing in the same settings have more frequent opportunities to observe and interact with each other, physicians practicing in different settings do not. As a result, they may assume that their personal experiences are representative of the experiences of other physicians in the CCOP. This would explain the high degree of correlation between individually referenced and group-referenced items measuring implementation climate perceptions among CCOP physicians.

By contrast, clinicians affiliated with children’s behavioral health agencies often practice in groups in single locations. They have more frequent opportunities to observe and interact with each other and, therefore, have a richer source of information from which to form perceptions of each other’s experiences. As a result, clinicians could more easily note differences between their own experience and those of other clinicians. The modest correlations between individually referenced and group-referenced items might indicate that, in this context, these items are measuring related, yet distinct constructs.

Although our study results do not provide clear-cut guidance, group-referenced items may be better suited than individually referenced items for measuring implementation climate and testing Klein and Sorra’s [1] theory of implementation effectiveness. Group-referenced items may be more likely to detect shared perceptions of climate, if they exist, because they direct respondents’ attention toward their social context rather than toward their personal situations [14]. Further, group-referenced climate items may also be more predictive of organizational outcomes than individually referenced items [42]. More theory development and research is needed, however, about the conditions under which individually referenced items and group-referenced items measure different constructs and, when they do, whether these differences matter.

### Measuring implementation climate as an organization-level construct

The results of Study 1 did not support the measurement of implementation climate as an organization-level construct. The results of Study 2 did. These divergent findings, again, may be explained by differences in context. The low intraclass correlation coefficients observed in Study 1 indicate that only a small percentage of the variation in physicians’ implementation climate perceptions occurred as a function of CCOP membership. Although the level of interrater agreement within CCOPs in some cases is high enough to justify aggregating individual perceptions to the organizational level, the low intraclass correlation coefficients imply that there is little reason to aggregate to the organizational level, since CCOPs did not vary much in implementation climate. Most CCOPs do not have formal expectations about the number of patients that physicians should enroll in clinical trials; those that do have fairly modest expectations. Although some physicians enjoy high levels of research support — namely those who practice in large groups or in ‘central’ locations within the CCOP — many do not have research staff on site or close by to help them enroll patients in trials. Given legal and ethical constraints, CCOPs cannot provide strong incentives or rewards to physicians for enrolling patients in trials. Recognition tends to be non-monetary, symbolic and social. The effects of such recognition may vary more at the individual level than between groups.

By contrast, the intraclass correlation coefficients observed in Study 2 indicate that a substantial percentage of variation in implementation climate perceptions occurred between implementation teams. Moreover, the interrater agreement within implementation teams was high enough to justify aggregating individual perceptions to the organizational level. Although fewer interrater agreement values achieved statistical significance in Study 2 than in Study 1, this may have reflected differences in average group size across the two studies. Implementation climate may have varied significantly between implementation teams because of compatibility of TF-CBT with organizational values and practices. TF-CBT is primarily intended for use in outpatient or community-based settings to treat children with trauma histories and their families. Although all Study 2 agencies provide children’s behavioral health services, there were several agencies that specialize in other areas (*i.e*., adult services), are generalists, or provide services in inpatient or residential settings, which would require substantial modifications to either administrative procedures, or TF-CBT in order to implement. In organizational settings with poor fit, there may be more implementation challenges, resistance, and limited or inconsistent messages about rewards, supports and expectations for implementation.

In summarizing the evidence for measuring implementation climate as an organization-level construct, we have given less weight to the within and between analysis, or WABA analysis, suggested by Van Miero and her colleagues as part of Step 3. WABA has become a less popular approach in the literature due in part to the growth of hierarchical linear modeling [34]. Given that WABA is based on one-way analysis of variance (ANOVA) and regression, WABA is also subject to the same set of assumptions, mainly homogeneity of variance, normality, statistical independence, and equal interval measurement [42]. An additional limitation of using WABA is that any restriction of between-groups variance on implementation climate may result in underestimation of within-cell agreement, and thus produce erroneous conclusions [43].

We also gave less weight to the within-group EFA (Step 5) for Study 2. There is ambiguity about what it means to have no meaningful factor structure, which allows researchers to conclude that there is no systematic measurement error present in the data. We were unclear as to whether our factor analysis for Study 2 exhibited no meaningful structure.

Since Klein and Sorra [1] conceived implementation climate as an organization-level construct, a proper test of their theory can occur only if climate perceptions are sufficiently shared among organizational members to justify aggregation of individual-level data to the organization-level of analysis. If, as in the case of Study 1, this condition is not met, researchers have two options. They could drop down a level of analysis, develop an individual-level analogue of implementation climate (much as researchers distinguish between organizational climate and psychological climate), and examine the association of this new construct with individual implementation effectiveness. Alternatively, they could measure the dispersion of implementation climate perceptions and examine whether organizations that exhibit greater variability in climate perceptions exhibit less effective implementation than those that exhibit greater consistency in climate perceptions. Either option would contribute to theory and research.

### Study limitations

The results of this study should be interpreted in light of several limitations. First, Study 2 had a smaller sample size in terms of the number of teams, the size of the teams, and the overall number of participating clinicians than Study 1. Although the number of observations was sufficient for the analyses conducted, larger sample sizes may yield more accurate parameter estimates, especially for the confirmatory factor analysis [44]. Also, the smaller team size may have contributed to a greater sense of shared experiences and perceptions and thus greater within group agreement. Notably, we did achieve high response rates, especially for physician and clinician participants. Therefore, our results are not likely to be subject to any systematic response biases. We should note, however, that in both studies, the individually referenced items appeared first in the survey; therefore, systematic bias due to ordering effects could have occurred.

Second, our study-specific findings have limited generalizability given our overall finding that contextual differences matter in how implementation climate should be conceptualized and measured. We can speculate that specific features of context such as the degree to which professionals work interdependently, physical proximity, and opportunities for interaction may influence whether implementation climate should be measured at the individual or organizational level. However, further inquiry across other practice contexts is necessary for determining the appropriate level at which implementation climate should be measured.

## Conclusions

Implementation climate is a global construct representing individual workers’ perceptions of how innovation use is rewarded, supported and expected. Although implementation climate can be considered an organizational level construct, its aggregation may depend entirely on context. In contexts where workers practice independently, implementation climate can be considered at the individual level. In comparison, in contexts where workers interact frequently and develop a shared perception, implementation climate can be a group-level context. It remains unclear, however, which types of survey question items should be used to capture group-level implementation climate (individual or group-referenced items). Therefore, researchers should engage in more systematic testing across various contexts to verify the observations in this paper and assess further the reliability and validity of the instrument we developed. For example, we need to test hypotheses about contextual variants, specifically those related to interactions among implementers: geographic proximity, cohesion, and task interdependence. In addition, researchers should seek more opportunities to validate this measure and understand how well it predicts and explains implementation effectiveness.

## Appendix

### Specific formulas for Steps 3 and 4

#### *Step 3. Variance within and between groups*

ICC(1) equals the correlation between the values of two randomly drawn individuals from a single randomly drawn group. ICC(2) represents the reliability of the group mean scores and varies as a function of ICC(1) and group size.

Values are obtained from a One-Way Analysis of Variance (ANOVA)

$$\mathrm{ICC}\left(1\right)=\frac{\mathrm{MSB}-\mathrm{MSW}}{\mathrm{MSB}+\left(N-1\right)\left(\mathrm{MSB}\right)}$$

$$\mathrm{ICC}\left(2\right)=\frac{\mathrm{MSB}-\mathrm{MSW}}{\mathrm{MSB}}$$

Where *MSB* = Mean Square Between; *MSW* = Mean Square Within; *N* = Number of Individuals in the Group

WABA I involves estimating between eta-correlations (*η*_*EX*_) and within eta-correlations (*η*_*WX*_) and testing for practical and statistical significance with E and F tests respectively. Practical significance based on 30° (group level if E ≥1.73, within-group level if E <0.577) or 15° test (group level if E ≥ 1.30, within-group level if E < 0.77). Statistical significance based on F test or 1/F test [34].

$$\begin{array}{l} \mathrm{WABA}\;I:\eta_{\mathrm{EX}}=\sqrt{\frac{SS_{B}}{SS_{T};}}\eta_{\mathrm{WX}}=\sqrt{\frac{SS_{W}}{SS_{T};}}E=\frac{\eta_{\mathrm{EX}}}{\eta_{\mathrm{WX}};}\\ \;F=E^{2}\frac{N-J}{J-1} \end{array}$$

Where *SS*_*B*_ = Sum of Squares Between; *SS*_*W*_ = Sum of Squares Within; *SS*_*T*_ = Sum of Squares Total; *N* = Number of Individuals; J = N_groups_

#### *Step 4. Agreement within teams*

A common measurement of interrater agreement is the *r*_*wg*(*j*)_ index for multiple items. It is obtained by comparing the observed variance in a group on a set of items to the variance that would be expected if group members would respond randomly. There are a number of limitations of using *r*_*wg*(*j*)_. To address some of these concerns, an alternative version named r*_wg(*j*)_ was developed. An important advantage of r*_wg(*j*)_ is that rating scales with large number of items do not result in overestimation of true agreement. For 5-point scales, -1.00 < r*_wg(*j*)_ < 1.00; r*_wg(*j*)_ = 0 in case of random response; and r*_wg(*j*)_ = 1 in case of maximum agreement. Dunlap et al. [39] provide r*_wg(*j*)_ significance levels for various combinations of sample size and number of categories.

$$r{*}_{\mathrm{wg}\left(j\right)}=1-\frac{{}_{\mathrm{Sxj}}^{-2}}{S_{\mathrm{EU}}^{2}}$$

Where ${}_{\mathrm{Sxj}}^{-2}$ = Mean of observed variances on J items; *S*_*EU*_ = Expected variance under uniform distribution = (A2-1)12 where A = Alternatives in response scale.

## Abbreviations

NCI: National Cancer Institute; CCOP: Community Clinical Oncology Program; TF-CBT: Trauma-focused cognitive behavioral therapy; NCI/DCP: National Cancer Institute Division of Cancer Prevention; ICC: Intraclass correlation coefficients; WABA: Within-and-between analysis; CFA: Confirmatory factor analysis; EFA: Exploratory factor analysis; CFI: Comparative fit indices; TLI: Tucker-Lewis fit indices; SRMR: Standard root mean square residuals; RMSEA: Root mean square errors of approximation.

## Competing interests

The authors declare that they have no competing interests.

## Authors’ contributions

SRJ helped carry out Study 1, performed all of the statistical analyses for Study 1, and drafted the manuscript. BJW conceived the overall study design and designed the study instrument. ACB carried out Study 2, and performed all of the statistical analyses for Study 2. All authors read and approved the final manuscript.
